# Games for the Men

**Published:** 1918-07-20

**Authors:** John Penoyre

**Affiliations:** 8 King's Bench Walk, Inner Temple, E.C. 4.


*To the Editor of* The Hospital.

### Games for the Men.

*To the Editor of* The Hospital.

Sir,—Can you yet find me a space to lay a plan, thrifty as becomes the times, before your readers? I am trying to keep up our issue of games for the armies abroad by combining and resorting all the waste games material in the country. Any odd stumps, balls, flannels, etc., come to their own in value when made up into sets; in fact, the odd parts of any game—indoor or out—can be given a new lease of usefulness in this way.

The men are most grateful for the games already sent, and most anxious for more. Please address any games, complete or incomplete, indoor or outdoor, to Sir Edward Ward, D.G.V.O., 45 Horseferry Road, S.W. 1.

Yours truly,
John Penoyre, C.B.E.

8 King's Bench Walk, Inner Temple, E.C. 4.
July 10, 1918.

